# Diagnostic Accuracy of Five Serologic Tests for *Strongyloides stercoralis* Infection

**DOI:** 10.1371/journal.pntd.0002640

**Published:** 2014-01-09

**Authors:** Zeno Bisoffi, Dora Buonfrate, Marco Sequi, Rojelio Mejia, Ruben O. Cimino, Alejandro J. Krolewiecki, Marco Albonico, Maria Gobbo, Stefania Bonafini, Andrea Angheben, Ana Requena-Mendez, José Muñoz, Thomas B. Nutman

**Affiliations:** 1 Center for Tropical Diseases (CTD), Sacro Cuore Hospital, Negrar, Verona, Italy; 2 Coordinating Resources to Assess and Improve Health Status of Migrants from Latin America (COHEMI) Project Study Group, European Commission, Health Cooperation Work Programme, FP7 (GA-261495); 3 Department of Public Health, IRCCS - Mario Negri Institute for Pharmacological Research, Milan, Italy; 4 National Institute of Allergy and Infectious Diseases (NIAID), National Institutes of Health (NIH), Bethesda, Maryland, United States of America; 5 Instituto de Investigaciones en Enfermedades Tropicales - Universidad Nacional de Salta/CONICET, Oran, Argentina; 6 Barcelona Centre for International Health Research (CRESIB, Hospital Clinic-Universitat de Barcelona), Barcelona, Spain; Texas Tech University Health Sciences Center, United States of America

## Abstract

**Background:**

The diagnosis of *Strongyloides stercoralis* (*S. stercoralis*) infection is hampered by the suboptimal sensitivity of fecal-based tests. Serological methods are believed to be more sensitive, although assessing their accuracy is difficult because of the lack of sensitivity of a fecal-based reference (“gold”) standard.

**Methods:**

The sensitivity and specificity of 5 serologic tests for *S. stercoralis* (in-house IFAT, NIE-ELISA and NIE-LIPS and the commercially available Bordier-ELISA and IVD-ELISA) were assessed on 399 cryopreserved serum samples. Accuracy was measured using fecal results as the primary reference standard, but also using a composite reference standard (based on a combination of tests).

**Results:**

According to the latter standard, the most sensitive test was IFAT, with 94.6% sensitivity (91.2–96.9), followed by IVD-ELISA (92.3%, 87.7–96.9). The most specific test was NIE-LIPS, with specificity 99.6% (98.9–100), followed by IVD-ELISA (97.4%, 95.5–99.3). NIE-LIPS did not cross-react with any of the specimens from subjects with other parasitic infections. NIE-LIPS and the two commercial ELISAs approach 100% specificity at a cut off level that maintains ≥70% sensitivity.

**Conclusions:**

NIE-LIPS is the most accurate serologic test for the diagnosis of *S. stercoralis* infection. IFAT and each of the ELISA tests are sufficiently accurate, above a given cut off, for diagnosis, prevalence studies and inclusion in clinical trials.

## Author Summary

The diagnosis of *Strongyloides stercorali*s infection is usually made by finding larvae of the parasite in the feces. The larval output is orders of magnitude lower than, say, the egg output of *Ancylostoma duodenale*, therefore the sensitivity of conventional techniques is poor. Sensitivity is enhanced by specific techniques, but the infection can still be missed. Several serologic methods (*Strongyloides* antibody detection in blood) are considered more sensitive, but they have been assessed so far with fecal tests as the gold standard, which is obviously unsatisfactory considering, precisely, their suboptimal sensitivity. Using a bank of sera from patients surely infected, not infected or doubtful, we assessed the accuracy of five different serologic tests also using a composite reference standard, obtained by combining the results of different tests. The recently developed NIE-LIPS resulted virtually 100% specific, with sensitivity >80%. Two commercially available ELISA tests were also highly specific above a given cut-off. Cross reactions with other parasitic infections were rarer than in previous studies. In conclusion, serologic tests are accurate tools, both for diagnostic purposes and for prevalence studies. Whether or not they can also be reliable markers of cure is currently under study.

## Introduction

*Strongyloides stercoralis* (*S. stercoralis*) is a nematode widely distributed all over the world, in areas where poor hygienic conditions permit the maintenance of its transmission. In the human host the infection is characterized by an autoinfective cycle, that can lead to life-long carriage of the parasite if left untreated [1]. For this reason, chronically infected patients are often found even in areas where transmission no longer occurs [2].

Chronic infection is often clinically silent. It is crucial, however, to detect and treat the infection in order to avoid the risk of the life-threatening complications (hyperinfection and dissemination) that can develop in the face of immunosuppression (e.g. underlying medical conditions and/or iatrogenic [steroids, other immunosuppressive agents]) [3].

Proper diagnostic testing is crucial both to identify *S. stercoralis*-infected individuals and to evaluate the prevalence of the infection among populations. One of the main problems with *S. stercoralis* is that its overall prevalence is probably underestimated [4], mostly due to the lack of sensitivity of fecal – based tests that are the most commonly used assessments for *S. stercoralis* infection. Serologic tests are also very useful, but their specificity is variable [5] and more difficult to assess because of the unreliability of the used reference test, i.e. microscopy. Discordant (fecal negative – serological positive) samples cannot be clearly defined. Furthermore, specificity is likely to be variable in different population groups and to be better in environments where other intestinal parasites are rare or absent, while sensitivity may be sub optimal in immunosuppressed patients [6].

An ideal diagnostic tool for *S. stercoralis* should have a very high sensitivity when used for screening (i.e. candidates for transplantation, chemotherapy, systemic corticosteroids) as well as to detect persistence of infection after treatment (therapeutic failure). Ideally the test should become negative or consistently show a marked decrease in titer in a predictable time after successful treatment. Although some studies document a decline of antibody titer after effective treatment, a clear cut-off value has yet to be defined [7], [8], [9], [10]. For a clinical trial, however, a very high specificity is needed in order to avoid inclusion of false positive subjects.

The main objective of the present study was to assess the accuracy of five serologic methods for the diagnosis of *S. stercoralis* infection in different patient populations. The serologic tools are intended for use both in highly endemic settings (screening of subjects at risk for complications, prevalence studies, clinical diagnosis in adequately equipped laboratories) and in areas of low or no endemicity (screening and diagnosis of immigrants, travelers, and autochthonous infection in elderly patients in countries previously endemic such as in Southern Europe).

## Methods

### Conduct of the study

The study was carried out in two reference laboratories for parasitic diseases (CTD Negrar - Verona, Italy and NIAID-NIH, Bethesda, US) by well-trained staff members. Samples were selected from a composite study population that is described in detail below. As fecal based methods are virtually 100% specific but lack sensitivity [10], [11], [12], a composite reference standard was also used (see below) as a suggested procedure for the evaluation of diagnostic tests when there is no gold standard [13], [14].

### Study design

The study was designed as a retrospective comparative diagnostic study on archived, anonymized serum samples. Sensitivity, specificity and positive and negative predictive values (PPV, NPV) of the index tests calculated against the primary reference standard (direct demonstration of *Strongyloides* larvae in stools by microscopy or culture) was used as the primary endpoint. A secondary endpoint was a test's sensitivity, specificity and predictive values when compared to a composite reference standard (as defined below).

### Study samples

The study was carried out on fully anonymized, coded serum samples already available at CTD that were selected randomly, within each study group outlined below. The archived specimens were kept frozen at −80°C from the day of the sample collection and tests were executed within 24 hours of unfreezing.

### Inclusion criteria

Serum specimens were selected from a composite patient population including:

**Group I** - Subjects of all ages with *S. stercoralis* larvae in fecal specimens, identified by microscopy and/or culture (primary reference standard)
**Group II -** Subjects with no previous exposure to *S. stercoralis*: healthy blood donors and patients of all ages, born and resident in non-endemic areas of Europe and with no travel history to endemic countries.
**Group III -** Subjects with potential, previous exposure to *S. stercoralis* but with negative fecal tests for strongyloidiasis:
a)  subjects routinely screened for parasites, with no known parasitic infections.
b)  patients with other parasitic infections (see below for details).

### Exclusion criteria

**Group I -** Hyperinfection syndrome (HS) or disseminated strongyloidiasis (DS). HIV patients with CD4+ cells <350/µL
**Group II -** History of farm work; age >50 years; previous residence in areas where *Strongyloides* transmission was known to occur in past decades
**Group III -** HIV patients with CD4+ cells <350/µL.

### Participant sampling and sample size

Based on an expected sensitivity of ∼90% and specificity of ∼95% (Group II) and 90% (Group III), sample sizes were calculated. Ultimately there were 114 in Group I (the *Strongyloides* infected group); 115 specimens for Group II and 170 for Group III. Within Group III b the parasitic infections diagnosed included: Schistosoma spp, *Trichinella spiralis*, *Toxocara canis*, *Fasciola hepatica*, *Echinococcus* granulosus, Hookworm, *Loa*, *Onchocerca volvulus*, *Mansonella perstans*, *Wuchereria bancrofti* and *Trypanosoma cruzi*. The study population is summarized in the STARD flow chart (Supporting Information Figure S1).

### Test methods

#### Primary reference standard

Direct detection of *S. stercoralis* larvae in stool, either through microscopy of at least three fecal samples after formol-ether concentration or Baermann, or stool agar/charcoal culture for *S. stercoralis*.

#### Composite reference standard

The subject classification to this purpose was: **Infected** (denominator for sensitivity): either a positive reference (fecal) test OR at least 3 positive results of the 5 serologic tests. **Not infected** (denominator for specificity): a negative reference (fecal) test AND <3 positive results out of the 5 serologic tests.

#### Index serologic tests

Index serologic tests included three non-commercial tests: IFAT (CTD) [15], NIE- ELISA [16] and NIE- LIPS [17] and 2 commercially available tests: Bordier ELISA (Bordier Affinity Products, Switzerland [18], batches 1120S and 1209S, expiry dates August 8^th^ 2013 and December 29^th^ 2013, respectively) and IVD-ELISA (SeroELISA *Strongyloides* IgG, IVD Research Carlsbad, CA [19], batch D2852, expiry date September 9^th^ 2013). Cutoffs for each test were pre-determined prior to testing.

A brief description of all the methods follows:

- IFAT (CTD – in house method): it detects IgG antibodies against *S. stercoralis*; for antigen preparation, intact *S. stercoralis* filariform larvae are obtained from a positive charcoal fecal culture, as it has been described previously [15]. Based on ROC analysis, samples with antibody titers ≥1∶20 were considered positives.
- NIE refers to a 31-kDa recombinant antigen derived from a *S. stercoralis* L3 cDNA library. NIE-based assays used in this trial were NIE- ELISA [16] and NIE- LIPS (Luciferase Immunoprecipitation System) [17]. For the LIPS assay, all data were corrected for background reactivity. Cut offs for negatives and positives were based on ROC analysis using sera from stool positive *Strongyloides*-infected patients and normal healthy controls as described [17]. For the NIE-ELISA, a standard curve was used and values (units/ml) interpolated from that standard curve [16]. ROC analyses performed previously were used to establish the negative and positive cutoffs for the NIE-ELISA. Cut-offs for NIE ELISA and NIE LIPS were ≥24.13 Units/ml and ≥1434 Relative Light Units (RLU), respectively.
- Bordier ELISA [18]: it detects Strongyloides IgG antibodies by using somatic antigens from larvae of *Strongyloides ratti*. According to the manufacturer's instructions, the result is positive when the absorbance of the analyzed sample is higher than the absorbance of the weak positive control (provided in the kit). For the study purpose, in order to be able to compare results from different sessions, we defined as positives samples with: absorbance of study sample/absorbance of weak positive serum≥1 (calculated value).
- IVD ELISA [19]: it detects Strongyloides IgG antibodies by using somatic antigens from larvae of *Strongyloides stercoralis*. Positive samples are defined by absorbance greater than 0.2 OD units. For the study purpose, absorbance of study sample/0.2≥1 (calculated value) was used as the cutoff.

### Number, training and expertise of the persons executing and reading the index tests and the reference standard

All index tests were executed by senior staff of the participating laboratories that are reference laboratories for parasitology in the respective countries. The (primary) reference standard tests had been carried out by senior staff of CTD lab who were in charge of fecal and blood parasite microscopy and stool culture for *Strongyloides*.

IFAT (involving subjective reading) was independently carried out by two senior staff members of CTD laboratory. Discordant results were read by a third senior staff of CTD. The two commercial ELISA tests were also performed at CTD.

NIE-ELISA and NIE-LIPS were performed at NIAID-NIH (the laboratory which developed the method) by a senior staff member with help from a member of the University of Salta (Argentina).

### Blinding

All sera were re-coded by persons not directly involved in the study. Laboratory staff involved in the study had no access to the source codes and therefore were blinded as of the results of the previous reference tests, as well as of the results of the other index tests.

### Statistical analysis

For both the primary and secondary endpoints, the sensitivity of each index test was calculated as the proportion of positive results over all positive samples at the primary reference test. It was further calculated for different cut-off levels for each test. Uncertainty was quantified using the 95% confidence intervals. Specificity was first calculated over all sera from patients of Group II (subjects with no previous exposure to *S. stercoralis*), then on the whole control group, as the proportion of negative results of the index tests. It was further calculated for pre defined cut-off levels of each test. Uncertainty was quantified the same way as above. The corresponding ROC curves were plotted for each of the five index tests. Predictive values (PPV, NPV) were then estimated for different, theoretical prevalence or pre-test probabilities, both for the dichotomous test results and for the different cut-off values according to the ROC curves. Confidence interval (95%) at different cut-off levels of the index tests were calculated by bootstrap method using 2000 samples. The Kappa test (with its 95% confidence interval) was performed to assess concordance between each index test and the primary and the composite reference standards, respectively, as well as between pairs of index tests. Cohen's Kappa measure was used to assess the agreement as follows: K<0, no agreement; K = 0–0.20, poor agreement; K = 0.21–0.40, fair agreement; K = 0.41–0.60, moderate agreement; K = 0.61–0.80, substantial agreement; and K = 0.81–1.00, nearly perfect agreement [20], [21]. Multiple logistic regression analysis was used to study the variation of main outcome variables according to potential predictor variables such as age; sex; continent of origin.

### Ethical issues

Samples were anonymously coded, unlinked from any information identifying the source individuals. Although the study was retrospective and no action on patients was involved, the study protocol was nevertheless submitted to the Ethics Committee of the Coordinating Site (Comitato Etico Provinciale di Verona) for approval. The latter acknowledged the study protocol and formally authorized the study (protocol n. 13286/09.11.01 of 24^th^ April, 2012).

## Results

Results are reported according to the STARD checklist (Supporting Information Table S1). The sample selection and the laboratory analyses were performed during the second semester of 2012. The median age of the whole study population was 39 y (range, 1–86, interquartile range 26), with no relevant differences between subgroups. As for the continent of origin, of the 114 patients of Group I, 56 (49%) came from Europe, 27 (24%) from Africa, 17 (15%) from Asia and 14 (12%) from South America. All 115 controls of Group II were from Europe, while, of the 170 controls of Group III, 51 (30%) were from Europe, 75 (44%) from Africa, 16 (9%) from Asia and 28 (17%) from South America.

The proportion of samples with at least 3 positive serologic tests within each study group is summarized in Table 1. There were 399 pre-treatment samples overall, 114 from subjects with a positive fecal test (the denominator for sensitivity based on the primary reference standard) and 285 from subjects with a negative fecal test (the denominator for specificity). Of the 114 *S. stercoralis* stool-positive subjects, 107 (93.9%) had at least three positive index tests.

**Table 1:** Proportion of samples with ≥3 positive serologic tests.

Study Group	≥3 positive serologic tests (%)	<3 positive serologic tests (%)	Total
**Group I** Samples from patients with Ss larvae in stool (denominator for sensitivity, primary reference standard)	107 (93.9)*	7 (6.1)*	114 (100)
**Group II** Samples from patients with no Ss larvae in stool, unexposed (denominator for specificity, primary reference standard)	0(0)	115 (100)∧	115 (100)
**Group III** Samples from patients with no Ss larvae in stool, exposed			
Group III a) Samples from patients with no Ss larvae in stool, exposed, no other parasites	11 (10.7)	92 (89.3)	103 (100)
Group III b) Samples from patients with no Ss larvae in stool, exposed, other parasites diagnosed	5 (7.5)	62 (92.5)	67 (100)
	16 (9.4)*	154 (90.6)∧	170 (100)
**TOTAL**	123 (30.8)	276 (69.2)	399 (100)

infected according to primary reference standard: N. 114.

not infected according to primary reference standard: N. 115+170 = 285.

infected according to composite reference standard: N. 130.

not infected according to composite reference standard: N. 269.

According to the composite reference standard, subjects classified as infected were 130 overall. Those classified as non-infected were 269 comprised of 115 from Group II and 154 from Group III. Of the latter, 62 subjects had another parasitic infection diagnosed (Group III b) and the remaining 92 had none (Group III a).

### Accuracy

The overall accuracy of index tests using the primary reference standard for sensitivity (subjects with positive fecal results), and control Group II for specificity (subjects with negative fecal results and no previous, potential exposure to *S. stercoralis*) is summarized in Table 2. The most sensitive test was the IFAT, with a sensitivity 93.9% (IC 89.5–98.3), followed by IVD ELISA with a sensitivity 91.2% (86.0–96.4). The most specific test was LIPS, with specificity 100%, followed by IVD ELISA with specificity 99.1% (97.4–100) and Bordier ELISA with specificity 98.3% (95.9–100).

**Table 2:** Test accuracy on samples from subjects with certain diagnosis (denominator for sensitivity: 114 subjects with Ss larvae in stool; denominator for specificity: 115 subjects with no Ss larvae in stool and no exposure).

TEST	Sensitivity (IC 95%)	Specificity (IC 95%)
NIE ELISA	75.4 (67.5–83.3)	94.8 (90.7–98.9)
NIE LIPS	85.1 (78.6–91.6)	100.0 (100.0–100.0)
IFAT	93.9 (89.5–98.3)	92.2 (87.3–97.1)
IVD ELISA	91.2 (86.0–96.4)	99.1 (97.4–100.0)
BORDIER ELISA	89.5 (83.8–95.1)	98.3 (95.9–100.0)

Of 170 subjects of Group III (negative fecal tests and potential previous exposure to *S. stercoralis*), 70 (41.2%) had at least one positive index test result (Table 3). Sixteen of the 70 specimens (22.9%) were probable true positives according to the composite reference standard, corresponding to 9.4% of probable cases detected by serology among subjects with a negative fecal result and potential exposure (Table 1). Therefore, 16 subjects initially classified as controls were subsequently found positive in at least 3 serologic tests; these were then included among cases when based on the composite reference standard. Five of the 16 samples were initially classified as Group III b, as they had other parasitic infections. Three of them were from Africa and had a filarial infection (with *Mansonella perstans*, *Loa* and *Onchocerca volvulus*, respectively), one from South America (with Chagas disease) and one from Europe (with *Toxocara*). Three samples were positive in 3 out of the 5 serologic tests for *Strongyloides*, while the remaining two (with *Onchocerca* and Chagas, respectively) were positive in all tests for *Strongyloides*. Eleven of the 16 samples were initially classified as belonging to Group III a, as they had no other parasitic infection diagnosed. Three of them were from Africa, 4 from South America, 4 from Europe. All control samples had been submitted for routine parasitological and serologic screening tests carried out at CTD. Five of these 11 tested positive in all serologic tests for *Strongyloides*, 5 were positive in 4 tests, and 1 was positive in 3 of the serological tests.

**Table 3:** Test results on samples from subjects with no Ss larvae in stool and risk of exposure (170 samples).

TEST	N positive (%)	N true positive (%) according to composite reference standard
NIE ELISA	24 (14.1%)	6 (25.0%)
NIE LIPS	13 (7.6%)	12 (92.3%)
IFAT	41 (24.1%)	16 (24.1%)
IVD ELISA	22 (12.9%)	16 (72.7%)
BORDIER ELISA	30 (17.6%)	16 (53.3%)
ANY TEST	70 (41.2%)	16 (22.9%)

The number of positive results in this group for the different tests and the proportion of true positives according to the composite reference standard is also reported in Table 3. NIE LIPS had the highest proportion of true positives (12/13 or 92.3%), followed by IVD ELISA (16/22 or 72.7%).

The test accuracy on the whole study population, according to both reference standards, is summarized in Table 4. Figures on sensitivity are similar for both reference standards. As far as specificity is concerned, NIE LIPS is, again, the most specific test (95.4% and 99.6% according to primary and composite reference standard, respectively), followed by the IVD ELISA (91.9% and 97.4%) and the Bordier ELISA (88.8% and 94.1%).

**Table 4:** Test accuracy on the whole study population.

a) Primary ref standard (114 infected, 285 uninfected)						
		IC95%			IC95%	
TEST	Sensitivity	LL	UL	Specificity	LL	UL
NIE ELISA	75.44	67.54	83.34	89.47	85.91	93.04
NIE LIPS	85.09	78.55	91.63	95.44	93.02	97.86
IFAT	93.86	89.45	98.27	82.46	78.04	86.87
IVD ELISA	91.23	86.04	96.42	91.93	88.77	95.09
BORDIER ELISA	89.47	83.84	95.11	88.77	85.11	92.44

b) Composite ref standard (130 infected, 269 uninfected)						
		IC95%			IC95%	
TEST	Sensitivity	LL	UL	Specificity	LL	UL
NIE ELISA	70.77	62.95	78.59	91.08	87.67	94.48
NIE LIPS	83.85	77.52	90.17	99.63	98.90	100.00
IFAT	94.62	90.74	98.50	87.36	83.39	91.33
IVD ELISA	92.31	87.73	96.89	97.40	95.50	99.30
BORDIER ELISA	90.77	85.79	95.75	94.05	91.23	96.88

### Cross reactions with other parasitic infections

Analyzing in detail Group III b (Table 5), LIPS did not cross-react with any of the specimens from subjects with other parasitic infections; IFAT with 7/62 (11.3%) specimens; IVD ELISA with 6/62 (9.7%) specimens; NIE-ELISA with 6/62 (9.7%) specimens; Bordier ELISA with 7/62 (11.3%) specimens. For 5/62 specimens (8.1%), two index tests gave false positive results, for 16/62 specimens (25.8%) only one test out of the five was false positive, while for the remaining 41 specimens (66.1%) all five tests were negative. As for the individual parasites, *Mansonella perstans* caused the highest proportion of false positive reactions.

**Table 5:** Cross reactions with other parasites (Group III b: 62 subjects).

	N false positives (FP)								
Parasite	IFAT	NIE LIPS	NIE ELISA	Bordier	IVD	Total FP samples	Total FP reactions	TOTAL reactions	% FP reactions
Echinococcus	1	0	0	0	0	1	1	**25**	4.0%
Fasciola	1	0	0	0	1	2	2	**25**	8.0%
Loa	1	0	1	0	1	2	3	**20**	15.0%
HW	1	0	0	0	0	1	1	**30**	4.0%
HW plus Trichinella	0	0	0	1	1	1	2	**5**	40.0%
Trichinella	1	0	0	1	1	3	3	**30**	10.0%
Mansonella	1	0	1	1	0	2	3	**20**	20.0%
Onchocerca	0	0	0	1	0	1	1	**10**	10.0%
Toxocara	0	0	1	1	0	1	2	**45**	4.4%
Chagas	0	0	1	0	0	1	1	**40**	2.5%
Schistosoma	1	0	2	1	1	5	5	**50**	10.0%
Wuchereria	0	0	0	1	1	1	2	**15**	13.3%
TOTAL FP	7	0	6	7	6	21	26	**315**	8.4%
**TOTAL samples**	**62**	**62**	**62**	**62**	**62**	**62**			
% false positives	11.3%	0%	9.7%	11.3%	9.7%	33.9%			

**Figure 1:**
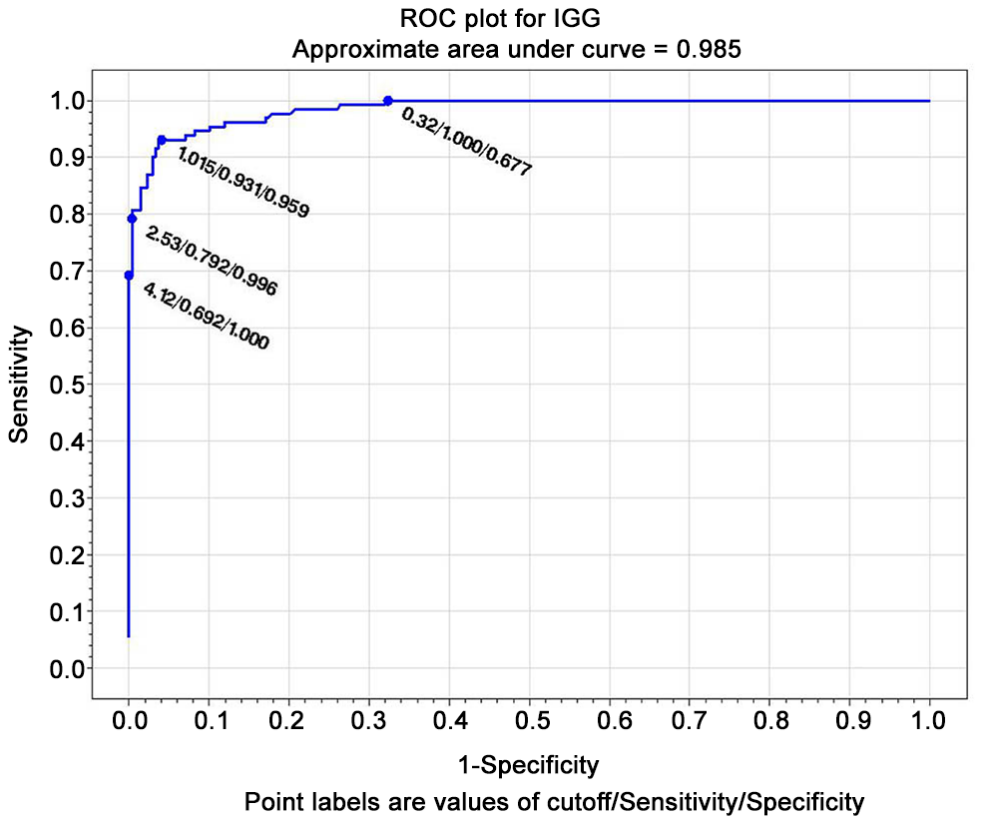
ROC curve for IVD ELISA (composite reference standard).

NB Total number of samples differs from Figure 1 (Group III b = 67), as 5 subjects were found positive in at least 3 serologic tests for *Strongyloides* and then classified as cases according to the composite reference standard.

#### ROC curves

**Figure 2:**
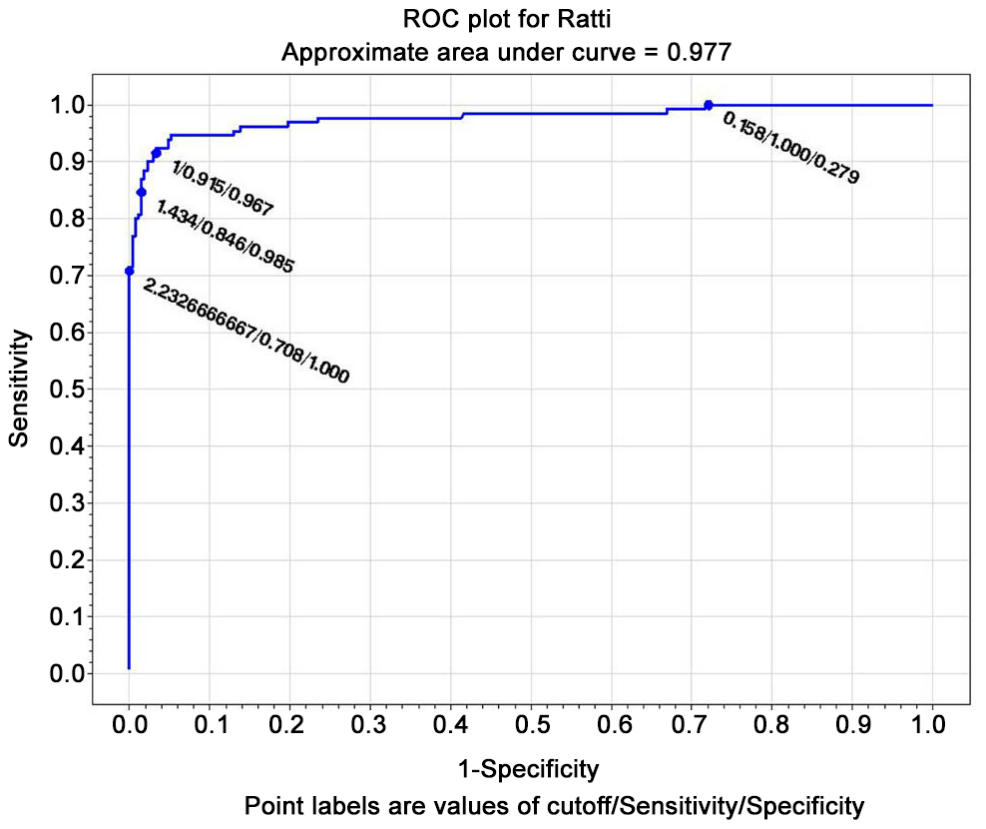
ROC curve for Bordier ELISA (composite reference standard).

**Figure 3:**
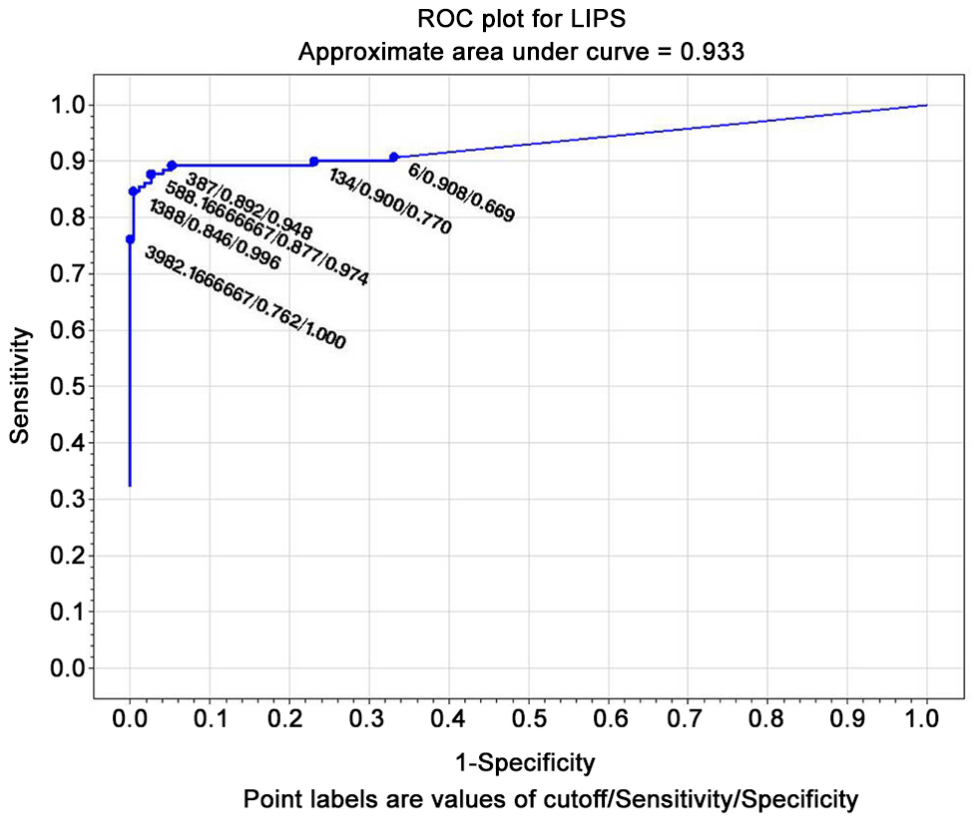
ROC curve for NIE-LIPS (composite reference standard).

**Figure 4:**
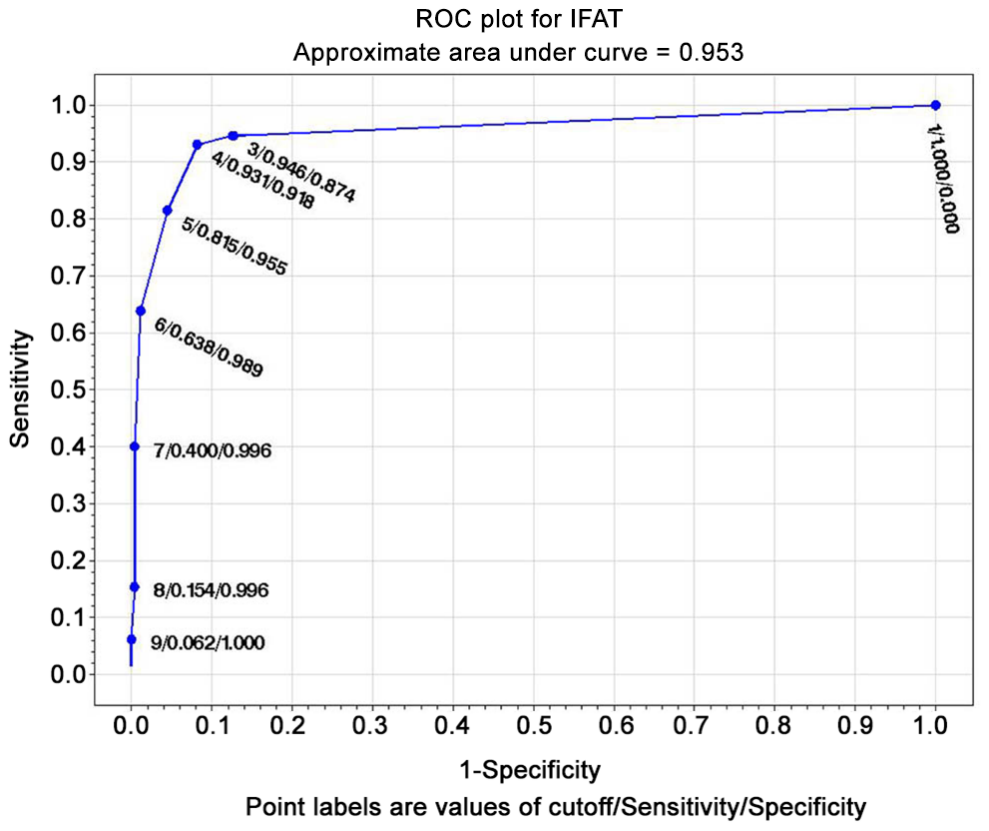
ROC curve for IFAT (composite reference standard) (numbers correspond to titers, 3 = 1/20 to 9 = 1/1280).

**Figure 5:**
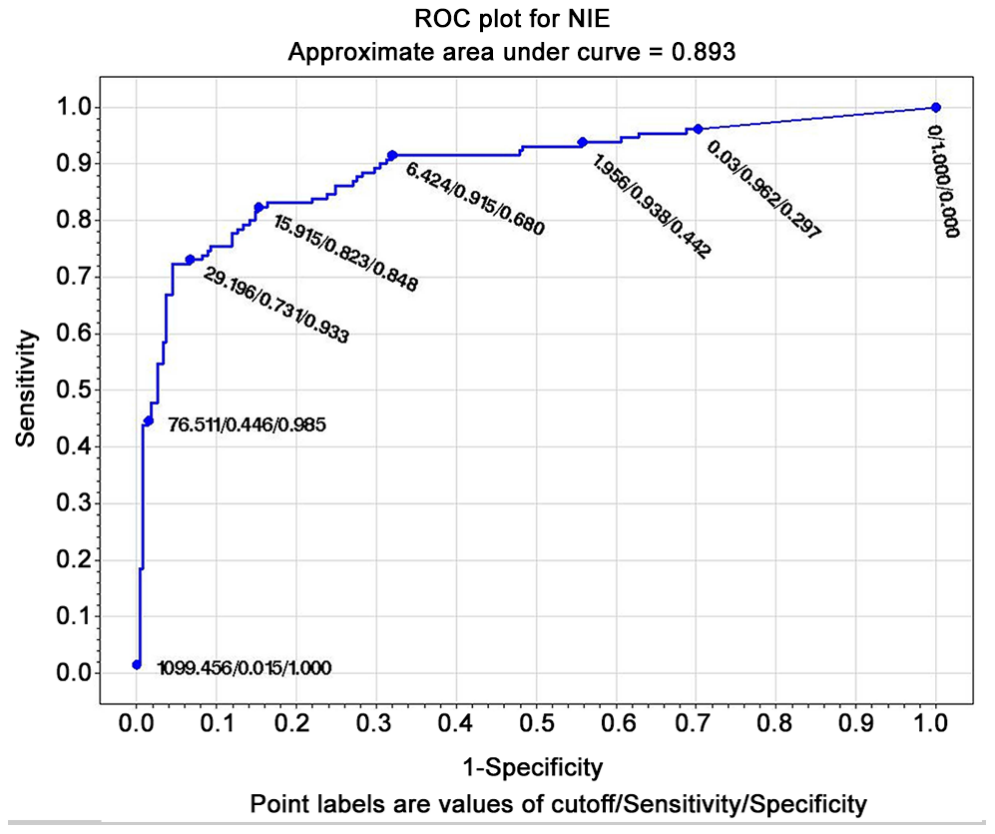
ROC curve for NIE-ELISA (composite reference standard).

The test with the widest area under the curve (at the composite reference standard) was IVD ELISA (0.985) (Figure 1), followed by Bordier ELISA (0.977) (Figure 2). LIPS (Figure 3) was virtually 100% specific at a cut-off value of 1388, at which sensitivity remained very high (84.6%). IVD reached the same specificity at a cut-off value of 2.53, at which sensitivity dropped to 79%. Bordier ELISA reached 100% specificity at a cut-off value of 2.23, corresponding to a sensitivity of 70%. IFAT (Figure 4) was 99% specific at cut-off 6 (1/160 titer) corresponding to a sensitivity of 64%. NIE ELISA (Figure 5) was 99% specific at cut-off 76.5 corresponding to a sensitivity of 45%. ROC curves for the five tests using the primary reference standard are reported in Supporting Information Figures S2, S3, S4, S5, S6. Accuracy for the different cut-off values of the tests is reported in detail in Supporting Information Table S2.

### Predictive values

Positive and negative predictive values (PPV, NPV) were estimated, based on the test accuracy and on different theoretical prevalence rates or pre-test probabilities (Supporting Information Table S3 and S4). Estimations were first made using the accuracy data summarized in Table 2, obtained from samples with an established diagnosis (Supporting Information Table S3). Further estimations were made using accuracy data summarized in Table 4 b, obtained by using the composite reference standard on the whole study population (Supporting Information Table S4). According to both standards, LIPS was the test with the highest PPV (100% and 69.6%, respectively) even at the lowest prevalence (1%), at which NPV was 99.8% (with both standards). IFAT and IVD ELISA had a NPV ≥99% up to a prevalence of 10%.

### Concordance

IVD ELISA showed the highest overall concordance with the composite reference standard (0.90, IC 0.86–0.95), followed by LIPS (0.87, IC 0.82–0.92), Bordier ELISA (0.84, IC 0.79–0.90), IFAT (0.78, IC 0.71–0.84) and NIE-ELISA (0.63, IC 0.55–0.72). The concordance between pairs of tests is reported in Supporting Information Table S5.

The highest concordance was between IVD ELISA and Bordier ELISA (0.83, IC 0.78–0.89), followed by that between IVD ELISA and LIPS. The lowest concordance was between IFAT and NIE ELISA (0.50, IC 0.41–0.59).

### Logistic regression

Age slightly correlated with infection, both using the primary reference standard (OR 1.020, IC 1.006–1.033) and the composite reference standard (OR 1.018, IC 1.006–1.031). Europe as the continent of origin as opposed to Asia correlated negatively with infection using the primary (OR 0.311, IC 0.131–0.737) as well as the composite reference standard (OR 0.326, IC 0.140–0.761).

## Discussion

With the present study we assessed the accuracy of five serologic tests for *S. stercoralis* not only against a primary reference standard (using fecal-based positivity), but also against a composite reference standard. This composite (potentially operational) reference standard allowed a more realistic classification of cases and controls, even though it carries a minor risk of misclassifying samples as false positives. All patients of Group II (most probably not exposed to *S. stercoralis*) consistently showed sero-negativity (i.e. not infected) according to the composite reference standard. If we consider only those with a positive fecal test as the denominator, almost 94% had at least three positive index tests (the required criterion to be classified as infected according to the composite reference standard).

### Test accuracy

The test with the highest specificity was NIE-LIPS, a test that virtually does not cause any false positive results. This can be an ideal test for clinical diagnosis, inclusion in clinical trials and prevalence studies, as its PPV is very high even at very low prevalence (1%). As far as predictive values are concerned (Tables S3 and S4), as ours was not a population-based study, we could only able make estimations based on different theoretical prevalences. The two reference standards used for accuracy did not appear to influence NPV (as sensitivity was very similar with both standards for each test), while PPV, not surprisingly, was higher when accuracy was estimated using the primary standard for cases and clearly unexposed (Group II) individuals as controls. For the purpose of screening of high risk groups (i.e., patients candidate to immunosuppressive treatment), the most suitable tests were IFAT and the two commercial ELISA, that maintain a NPV close to or higher than 99% up to a prevalence of 10%, regardless the reference standard used for the estimations. For higher prevalence, no test would safely exclude the infection, and two alternative options could be a screening with two different tests, or a presumptive treatment.

### Cross reactions

LIPS, challenged with specimens from patients with several different parasitic infections, confirms, even in this group, its excellent specificity. The other tests gave a variable proportion of false positive results, but less than previously reported (all previous studies relied exclusively on fecal tests as reference standard).

### ROC analysis, choice of a test cut-off for inclusion in clinical trial

ROC analysis (Figures 1–5) provides indications of a suitable cut-off for each test, in order to reach or approach a 100% certainty of infection for positive results, obviously at the expenses of some loss of sensitivity. We can thus propose a suitable cut-off level for each test for inclusion in a clinical trial (when a certainty or at least a very high probability of infection is required). NIE LIPS appears to be the best test for this purpose. IFAT loses sensitivity when gaining specificity at the optimal cut off for inclusion (titer 1/160). The two commercially available ELISA tests (IVD and Bordier, in this order) showed reliable results in terms of accuracy and can also be used for inclusion in trials at a cut-off of, respectively, ≥2.5, ≥2.2 at which they approach 100% specificity, while maintaining >70% sensitivity. Such standard and available tests could be used both in clinical and public health practices. It must be mentioned, however, that tests based on crude antigen may be difficult to ensure optimal reproducibility among different batches. We strongly recommend laboratories using these tests to put into place clear quality control methods.

### Study limitations

This study has the potential limitations inherent to a retrospective study design. Some quite relevant data were missing for some of the control subjects (i.e. the continent of exposure when/if it did not coincide with the continent of origin). Moreover, as parasitological methods are not 100% sensitive, also for other parasitic infections, it may well be that some infections were missed in control subjects exposed, which may have caused cross reactivity. While we believe that subjects were better classified using the composite reference standard, we cannot exclude a possible misclassification of some of them.

### Conclusion and further research needs

The issue of serology as a marker of cure remains an open question. If we were to rely on fecal-based diagnosis alone, we may wrongly consider cured a patient whose parasite load after treatment is too low to be detected. Thus, an evaluation of serologic tests to assess cure is currently underway. A prospective study that will include PCR on fecal samples is also planned. The ultimate aim is to identify the optimal diagnostic strategy for *S. stercoralis* for clinical and epidemiological purposes.

## Supporting Information

Figure S1 STARD flow chart.
(DOC)

Figure S2 ROC curve for IVD ELISA (primary reference standard).
(JPG)

Figure S3 ROC curve for Bordier ELISA (primary reference standard).
(JPG)

Figure S4 ROC curve for NIE-LIPS (primary reference standard).
(JPG)

Figure S5 ROC curve for IFAT (primary reference standard) (numbers correspond to titers, 3 = 1/20 to 9 = 1/1280).
(JPG)

Figure S6 ROC curve for NIE-ELISA (primary reference standard).
(JPG)

Table S1 STARD checklist for reporting of studies of diagnostic accuracy.
(DOC)

Table S2 Test accuracy (composite reference standard) at different cut-off levels of the index tests.
(DOC)

Table S3 Positive and negative predictive values (PPV, NPV) for different theoretical prevalence levels.
(DOC)

Table S4 Positive and negative predictive values (PPV, NPV) for different theoretical prevalence levels.
(DOC)

Table S5 Concordance between pairs of index tests (Kappa test).
(DOC)
