# Supplementary figures and images for: Diagnostic Accuracy of Five Serologic Tests for Strongyloides stercoralis Infection
Source: PLoS Negl Trop Dis. 2014 Jan 9;8(1):e2640. doi: 10.1371/journal.pntd.0002640 (PMC3890421; doi:10.1371/journal.pntd.0002640)

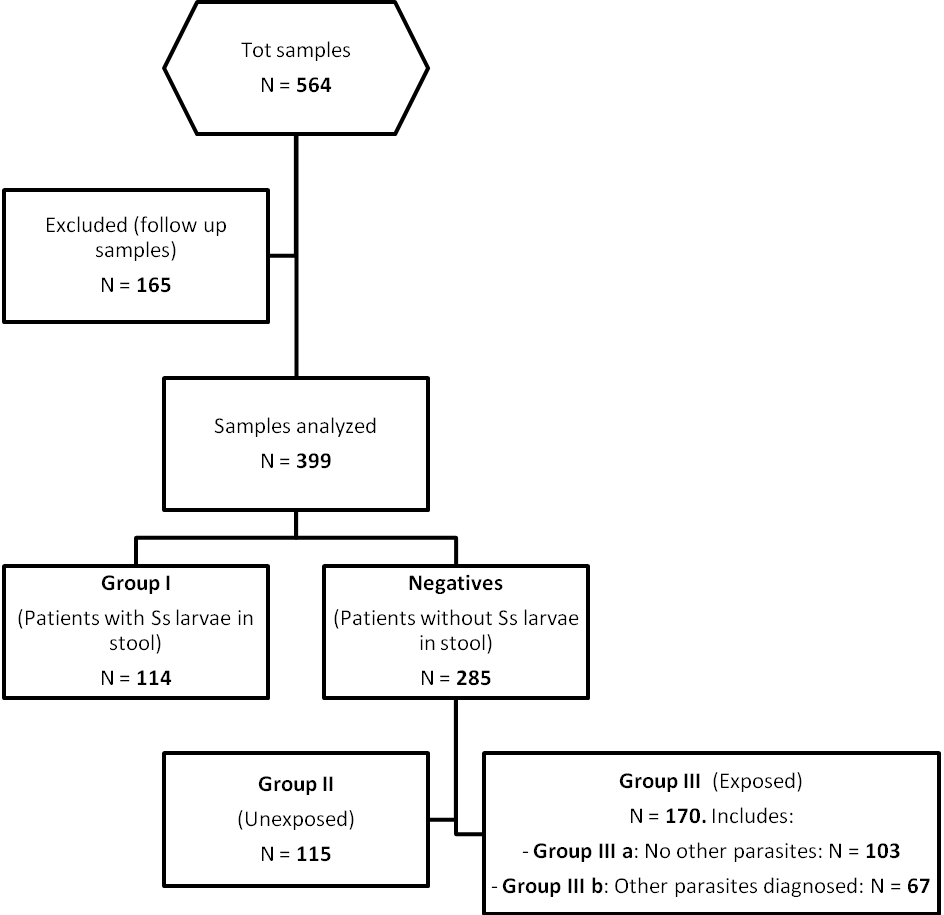

Supplement: Figure S1 — STARD flow chart. (DOC) [file pntd.0002640.s001.doc]

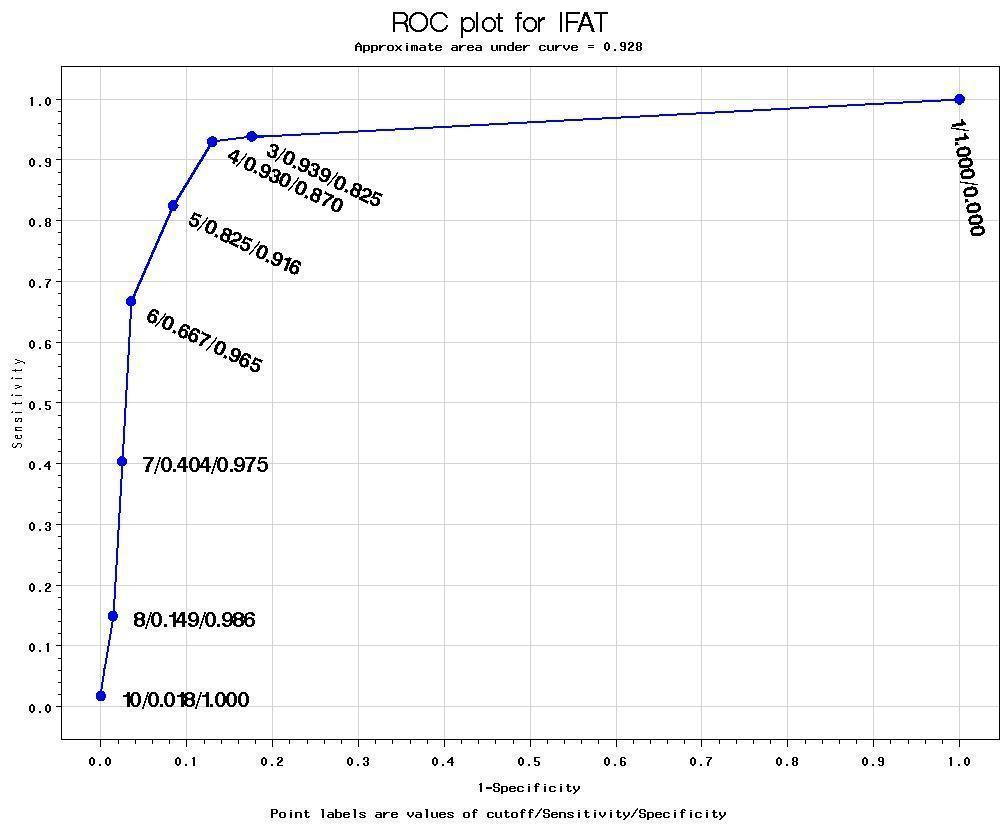

Supplement: Figure S2 — ROC curve for IVD ELISA (primary reference standard). (JPG) [file pntd.0002640.s002.jpg]

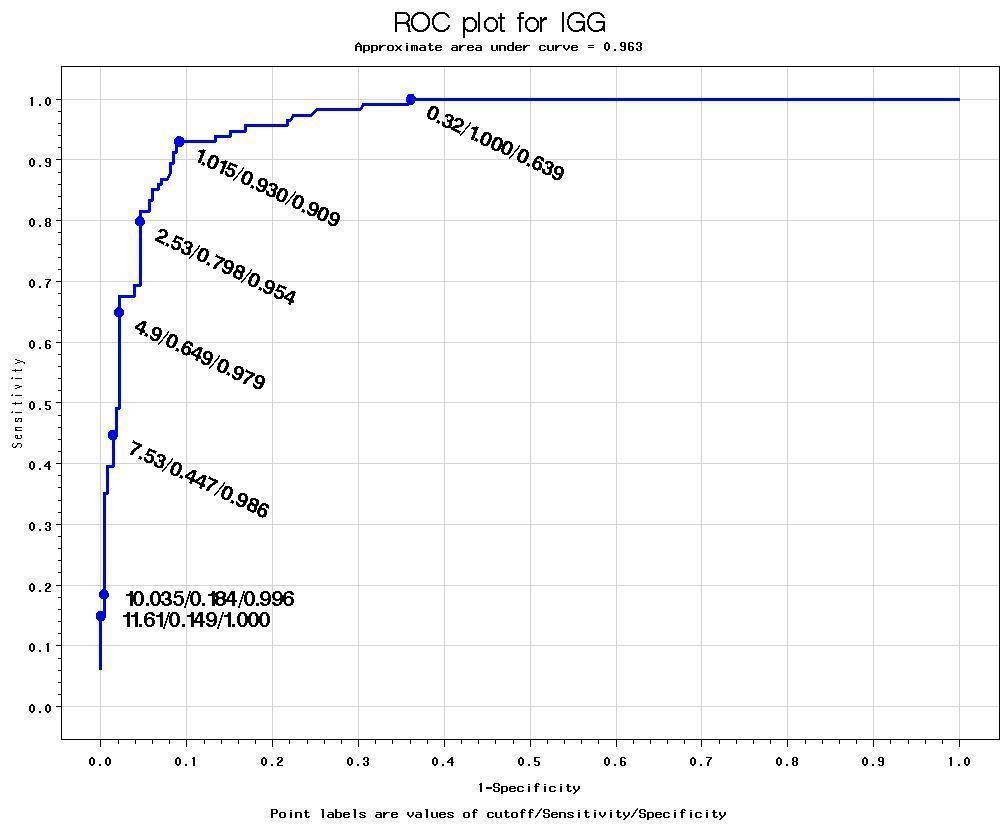

Supplement: Figure S3 — ROC curve for Bordier ELISA (primary reference standard). (JPG) [file pntd.0002640.s003.jpg]

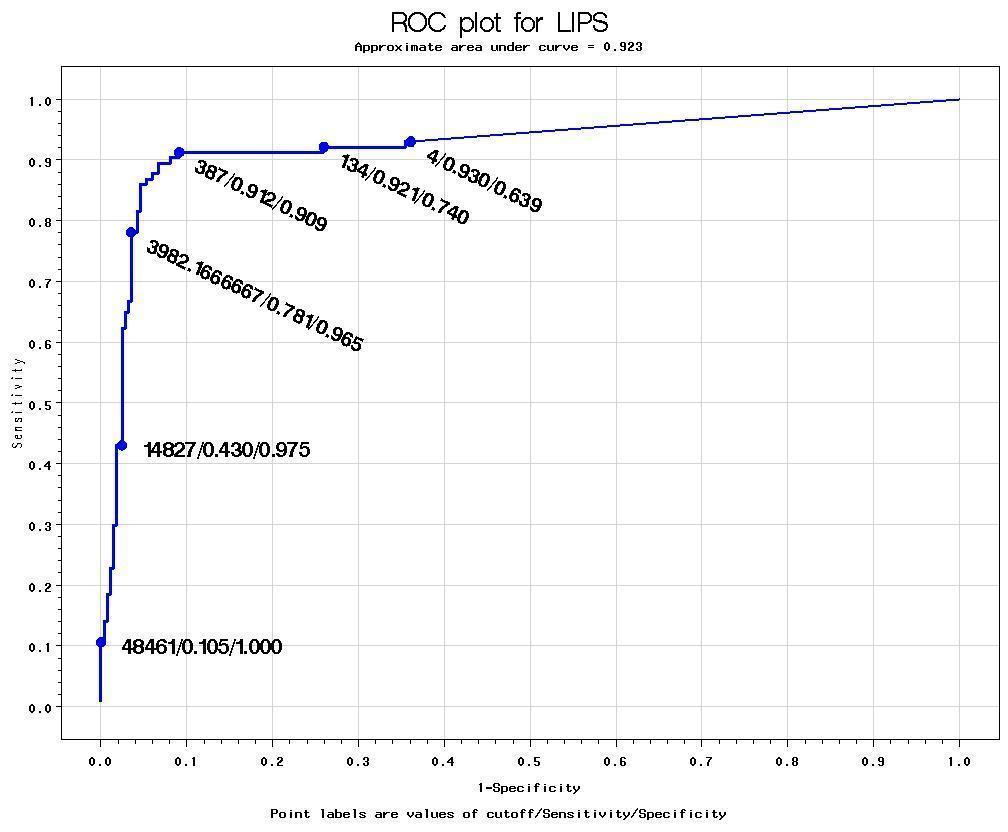

Supplement: Figure S4 — ROC curve for NIE-LIPS (primary reference standard). (JPG) [file pntd.0002640.s004.jpg]

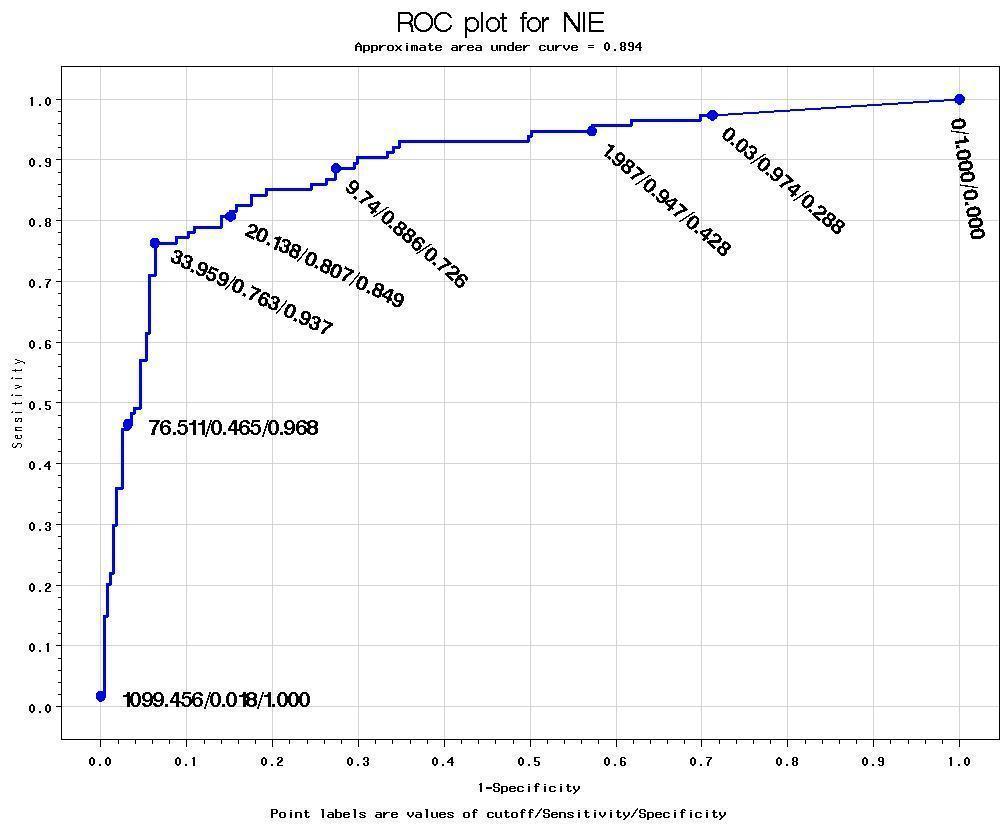

Supplement: Figure S5 — ROC curve for IFAT (primary reference standard) (numbers correspond to titers, 3 = 1/20 to 9 = 1/1280). (JPG) [file pntd.0002640.s005.jpg]

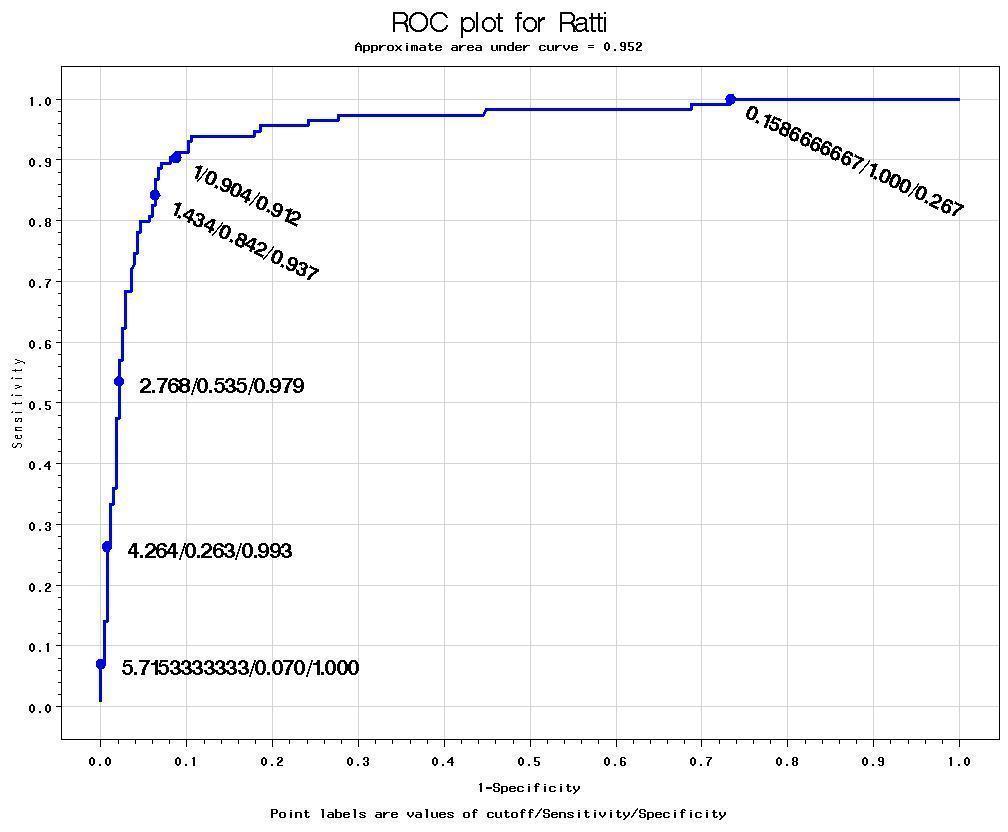

Supplement: Figure S6 — ROC curve for NIE-ELISA (primary reference standard). (JPG) [file pntd.0002640.s006.jpg]
