# Supplementary material for: Diagnostic Accuracy of Five Serologic Tests for Strongyloides stercoralis Infection
Source: PLoS Negl Trop Dis. 2014 Jan 9;8(1):e2640. doi: 10.1371/journal.pntd.0002640 (PMC3890421; doi:10.1371/journal.pntd.0002640)
Supplement: Table S2 — Test accuracy (composite reference standard) at different cut-off levels of the index tests. (DOC) [file pntd.0002640.s008.doc]

**Table S2. Test accuracy (composite reference standard) at different cut-off levels of the index tests**

|  |  |  | **IC95%** | |  | **IC95%** | |
| --- | --- | --- | --- | --- | --- | --- | --- |
| **TEST** | **Cutoff** | **Sensitivity** | **LL** | **UL** | **Specificity** | **LL** | **UL** |
| **NIE-ELISA** | 0,03 | 96,15 | 92,31 | 99,23 | 29,74 | 24,54 | 35,32 |
|  | 1,96 | 93,85 | 89,23 | 97,69 | 44,24 | 38,29 | 50,19 |
|  | 6,42 | 91,54 | 86,15 | 96,15 | 68,03 | 62,44 | 73,61 |
|  | 15,92 | 82,31 | 75,38 | 88,46 | 84,76 | 80,67 | 88,85 |
|  | 29,20 | 73,08 | 65,38 | 80,77 | 93,31 | 90,33 | 95,91 |
|  | 76,51 | 44,62 | 36,15 | 53,08 | 98,51 | 97,03 | 99,63 |
| **NIE-LIPS** | 6,00 | 90,77 | 85,38 | 95,38 | 66,91 | 61,34 | 72,86 |
|  | 134,00 | 90 | 84,62 | 94,62 | 76,95 | 71,75 | 81,78 |
|  | 387,00 | 89,23 | 83,08 | 94,62 | 94,8 | 91,82 | 97,4 |
|  | 588,17 | 87,7 | 80,77 | 92,31 | 97,4 | 95,17 | 99,26 |
|  | 1388,00 | 84,62 | 77,69 | 90,77 | 99,63 | 98,88 | 100 |
|  | 3982,17 | 76,2 | 67,69 | 82,31 | 100 | 100 | 100 |
| **IFAT (titer)** |  |  |  |  |  |  |  |
|  | 20 | 94,62 | 90,77 | 98,46 | 87,36 | 83,27 | 91,08 |
|  | 40 | 93,08 | 88,46 | 96,92 | 91,82 | 88,1 | 94,8 |
|  | 80 | 81,54 | 74,62 | 87,69 | 95,54 | 92,94 | 97,77 |
|  | 160 | 63,85 | 55,38 | 71,54 | 98,88 | 97,4 | 100 |
|  | 320 | 40 | 31,54 | 48,46 | 99,63 | 98,88 | 100 |
|  | 640 | 15,38 | 10 | 22,31 | 99,63 | 98,88 | 100 |
|  | 1280 | 6,154 | 2,308 | 10,77 | 100 | 100 | 100 |
| **IVD ELISA** | 0,32 | 100 | 100 | 100 | 67,66 | 61,71 | 73,23 |
|  | 1,02 | 93,08 | 88,46 | 96,92 | 95,91 | 93,31 | 98,14 |
|  | 2,53 | 79,23 | 72,31 | 86,15 | 99,63 | 98,88 | 100 |
|  | 4,12 | 69,23 | 60,77 | 76,92 | 100 | 100 | 100 |
| **BORDIER ELISA** | 0,16 | 100 | 100 | 100 | 27,88 | 22,68 | 33,09 |
|  | 1 | 91,54 | 86,92 | 96,15 | 96,65 | 94,42 | 98,51 |
|  | 1,43 | 84,62 | 78,46 | 90,77 | 98,51 | 96,65 | 99,63 |
|  | 2,23 | 70,8 | 62,31 | 77,69 | 100 | 100 | 100 |
