# Supplementary material for: Diagnostic Accuracy of Five Serologic Tests for Strongyloides stercoralis Infection
Source: PLoS Negl Trop Dis. 2014 Jan 9;8(1):e2640. doi: 10.1371/journal.pntd.0002640 (PMC3890421; doi:10.1371/journal.pntd.0002640)
Supplement: Table S3 — Positive and negative predictive values (PPV, NPV) for different theoretical prevalence levels. (DOC) [file pntd.0002640.s009.doc]

| **Table S3. Positive and negative predictive values (PPV, NPV) for different theoretical prevalence levels.**  Estimations are based on accuracy data obtained from subjects with certain diagnosis (Table 2) | | | | | | | | |
| --- | --- | --- | --- | --- | --- | --- | --- | --- |
| **Test** | **Prevalence** | **Sensitivity** | **Specificity** | **PPV** | **NPV** |  |  |  |
| **BORDIER ELISA** | **1,0%** | **89,5%** | **98,3%** | **34,7%** | **99,9%** |  |  |  |
|  | 5,0% | **89,5%** | **98,3%** | 73,5% | 99,4% |  |  |  |
|  | 10,0% | **89,5%** | **98,3%** | 85,4% | 98,8% |  |  |  |
|  | 20,0% | **89,5%** | **98,3%** | 92,9% | 97,4% |  |  |  |
|  | 30,0% | **89,5%** | **98,3%** | 95,8% | 95,6% |  |  |  |
| **IVD ELISA** | **1,0%** | **91,2%** | **99,1%** | **50,6%** | **99,9%** |  |  |  |
|  | 5,0% | **91,2%** | **99,1%** | 84,2% | 99,5% |  |  |  |
|  | 10,0% | **91,2%** | **99,1%** | 91,8% | 99,0% |  |  |  |
|  | 20,0% | **91,2%** | **99,1%** | 96,2% | 97,8% |  |  |  |
|  | 30,0% | **91,2%** | **99,1%** | 97,8% | 96,3% |  |  |  |
| **NIE ELISA** | **1,0%** | **75,4%** | **94,8%** | **32,3%** | **99,8%** |  |  |  |
|  | 5,0% | **75,4%** | **94,8%** | 71,3% | 98,7% |  |  |  |
|  | 10,0% | **75,4%** | **94,8%** | 84,0% | 97,3% |  |  |  |
|  | 20,0% | **75,4%** | **94,8%** | 92,2% | 94,1% |  |  |  |
|  | 30,0% | **75,4%** | **94,8%** | 95,3% | 90,3% |  |  |  |
| **IFAT** | **1,0%** | **93,9%** | **92,2%** | **10,8%** | **99,9%** |  |  |  |
|  | 5,0% | **93,9%** | **92,2%** | 38,8% | 99,7% |  |  |  |
|  | 10,0% | **93,9%** | **92,2%** | 57,2% | 99,3% |  |  |  |
|  | 20,0% | **93,9%** | **92,2%** | 75,1% | 98,4% |  |  |  |
|  | 30,0% | **93,9%** | **92,2%** | 83,8% | 97,2% |  |  |  |
| **LIPS** | **1,0%** | **85,1%** | **100%** | **100%** | **99,9%** |  |  |  |
|  | 5,0% | **85,1%** | **100%** | **100%** | 99,2% |  |  |  |
|  | 10,0% | **85,1%** | **100%** | **100%** | 98,4% |  |  |  |
|  | 20,0% | **85,1%** | **100%** | **100%** | 96,4% |  |  |  |
|  | 30,0% | **85,1%** | **100%** | **100%** | 94,0% |  |  |  |
