# Supplementary material for: Diagnostic Accuracy of Five Serologic Tests for Strongyloides stercoralis Infection
Source: PLoS Negl Trop Dis. 2014 Jan 9;8(1):e2640. doi: 10.1371/journal.pntd.0002640 (PMC3890421; doi:10.1371/journal.pntd.0002640)
Supplement: Table S4 — Positive and negative predictive values (PPV, NPV) for different theoretical prevalence levels. (DOC) [file pntd.0002640.s010.doc]

| **Table S4. Positive and negative predictive values (PPV, NPV) for different theoretical prevalence levels**  Estimations are based on accuracy data obtained from composite reference standard on the whole study population (Table 4 b) | | | | | | | | |
| --- | --- | --- | --- | --- | --- | --- | --- | --- |
| **Test** | **Prevalence** | **Sensitivity** | **Specificity** | **PPV** | **NPV** |  |  |  |
| **BORDIER ELISA** | **1,0%** | **90,8%** | **94,1%** | **13,4%** | **99,9%** |  |  |  |
|  | 5,0% | 90,8% | 94,1% | 44,5% | 99,5% |  |  |  |
|  | 10,0% | 90,8% | 94,1% | 62,9% | 98,9% |  |  |  |
|  | 20,0% | 90,8% | 94,1% | 79,2% | 97,6% |  |  |  |
|  | 30,0% | 90,8% | 94,1% | 86,7% | 96,0% |  |  |  |
| **IVD ELISA** | **1,0%** | **92,3%** | **97,4%** | **26,4%** | **99,9%** |  |  |  |
|  | 5,0% | 92,3% | 97,4% | 65,1% | 99,6% |  |  |  |
|  | 10,0% | 92,3% | 97,4% | 79,8% | 99,1% |  |  |  |
|  | 20,0% | 92,3% | 97,4% | 89,9% | 98,1% |  |  |  |
|  | 30,0% | 92,3% | 97,4% | 93,8% | 96,7% |  |  |  |
| **NIE ELISA** | **1,0%** | **70,8%** | **91,1%** | **7,4%** | **99,7%** |  |  |  |
|  | 5,0% | 70,8% | 91,1% | 29,5% | 98,3% |  |  |  |
|  | 10,0% | 70,8% | 91,1% | 46,9% | 96,6% |  |  |  |
|  | 20,0% | 70,8% | 91,1% | 66,5% | 92,6% |  |  |  |
|  | 30,0% | 70,8% | 91,1% | 77,3% | 87,9% |  |  |  |
| **IFAT** | **1,0%** | **94,6%** | **87,4%** | **7,0%** | **99,9%** |  |  |  |
|  | 5,0% | 94,6% | 87,4% | 28,3% | 99,7% |  |  |  |
|  | 10,0% | 94,6% | 87,4% | 45,4% | 99,3% |  |  |  |
|  | 20,0% | 94,6% | 87,4% | 65,2% | 98,5% |  |  |  |
|  | 30,0% | 94,6% | 87,4% | 76,2% | 97,4% |  |  |  |
| **LIPS** | **1,0%** | **83,9%** | **99,6%** | **69,6%** | **99,8%** |  |  |  |
|  | 5,0% | 83,9% | 99,6% | 92,3% | 99,2% |  |  |  |
|  | 10,0% | 83,9% | 99,6% | 96,2% | 98,2% |  |  |  |
|  | 20,0% | 83,9% | 99,6% | 98,3% | 96,1% |  |  |  |
|  | 30,0% | 83,9% | 99,6% | 99,0% | 93,5% |  |  |  |
