# Supplementary material for: Diagnostic Accuracy of Five Serologic Tests for Strongyloides stercoralis Infection
Source: PLoS Negl Trop Dis. 2014 Jan 9;8(1):e2640. doi: 10.1371/journal.pntd.0002640 (PMC3890421; doi:10.1371/journal.pntd.0002640)
Supplement: Table S5 — Concordance between pairs of index tests (Kappa test). (DOC) [file pntd.0002640.s011.doc]

**Table S5. Concordance between pairs of index tests (Kappa test)**

| **TEST** | **NIE** | | | **LIPS** | | | **IFAT** | | | **IVD** | | |
| --- | --- | --- | --- | --- | --- | --- | --- | --- | --- | --- | --- | --- |
|  | **Concordance** | **IC 95%** | | **Concordance** | **IC 95%** | | **Concordance** | **IC 95%** | | **Concordance** | **IC 95%** | |
| **NIE** | \ | \ | \ |  |  |  |  |  |  |  |  |  |
| **LIPS** | 0,64 | 0,56 | 0,73 | \ | \ | \ |  |  |  |  |  |  |
| **IFAT** | **0,50** | 0,41 | 0,59 | 0,70 | 0,62 | 0,77 | \ | \ | \ |  |  |  |
| **IVD** | 0,58 | 0,49 | 0,67 | **0,81** | 0,75 | 0,88 | 0,74 | 0,67 | 0,81 | \ | \ | \ |
| **BORDIER** | 0,57 | 0,48 | 0,66 | 0,78 | 0,71 | 0,84 | 0,69 | 0,62 | 0,77 | **0,83** | 0,78 | 0,89 |
